# Comparing the backfilling of mesoporous titania thin films with hole conductors of different sizes sharing the same mass density

**DOI:** 10.1107/S2052252520000913

**Published:** 2020-02-12

**Authors:** Raphael S. Märkl, Nuri Hohn, Emanuel Hupf, Lorenz Bießmann, Volker Körstgens, Lucas P. Kreuzer, Gaetano Mangiapia, Matthias Pomm, Armin Kriele, Eric Rivard, Peter Müller-Buschbaum

**Affiliations:** a Physik-Department, Lehrstuhl für Funktionelle Materialien, Technische Universität München, James-Franck-Strasse 1, Garching 85748, Germany; b Department of Chemistry, University of Alberta, 11227 Saskatchewan Dr, Edmonton, Alberta T6G 2G2, Canada; c Helmholtz Zentrum Geesthacht at Heinz Maier-Leibnitz-Zentrum, Lichtenbergstr. 1, Garching 85748, Germany; d Heinz Maier-Leibnitz-Zentrum, Lichtenbergstr. 1, Garching 85748, Germany

**Keywords:** backfilling, mesoporous titania, semiconducting polymers, tellurophene, grazing incidence small-angle neutron scattering

## Abstract

Efficient infiltration of a mesoporous titania matrix with conducting organic polymers or small molecules is one key challenge to overcome for hybrid photovoltaic devices. A quantitative analysis of the backfilling efficiency with time-of-flight grazing incidence small-angle neutron scattering (ToF-GISANS) and scanning electron microscopy (SEM) measurements is presented. Differences in the morphology due to the backfilling of mesoporous titania thin films are compared for the macromolecule poly[4,8-bis­(5-(2-ethyl­hexyl)­thio­phen-2-yl)benzo[1,2-*b*;4,5-*b*′]di­thio­phene-2,6-diyl-*alt*-(4-(2-ethyl­hexyl)-3-fluoro­thieno[3,4-*b*]thio­phene-)-2-carboxyl­ate-2-6-diyl)] (PTB7-Th) and the heavy-element containing small molecule 2-pinacol­boronate-3-phenyl­phen­anthro[9,10-*b*]telluro­phene (PhenTe-BPinPh). Hence, a 1.7 times higher backfilling efficiency of almost 70% is achieved for the small molecule PhenTe-BPinPh compared with the polymer PTB7-Th despite sharing the same volumetric mass density. The precise characterization of structural changes due to backfilling reveals that the volumetric density of backfilled materials plays a minor role in obtaining good backfilling efficiencies and interfaces with large surface contact.

## Abstract 2

Mesoporous titania thin films backfilled with the conjugated polymer PTB7-Th or the small-molecule PhenTe-BPinPh have been studied as novel materials for hybrid photovoltaics. Together with observed structural changes due to backfilling, volumetric mass density can be excluded in determining factors that influence backfilling efficiency for solar cell applications.

## 1. Introduction   

**Figure 1:**
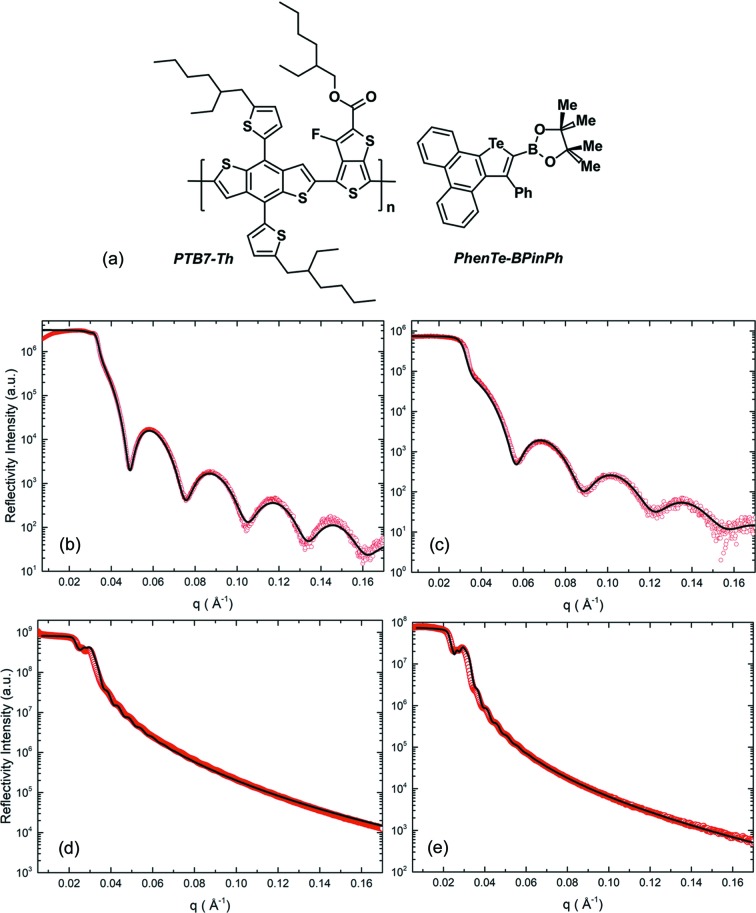
(*a*) Structures of PTB7-Th and PhenTe-BPinPh. (*b*)–(*e*) XRR curves of PTB7-Th spin-coated at (*b*) 1000 rpm on glass and (*c*) 1500 rpm on silicon with fit (solid line), and of PhenTe-BPinPh spin-coated at 1000 rpm on silicon (*d*) with subsequent SVA and (*e*) without SVA.

Creating maximum surface contact between functional materials is one of the most challenging and vital aspects of functional interface design (Graetzel *et al.*, 2012 ▸). Especially in photovoltaic applications, a delicate balance between maximizing the active material volume while minimizing charge carrier pathway distances and preventing loss mechanisms such as incomplete backfilling or short-circuit channels must be found (Snaith *et al.*, 2008 ▸). The formation of bulk heterojunction morphologies has proven to be a successful approach in achieving these properties with several fabrication methods existing, either by direct blending of two materials, addition of nanoparticles to a polymer solution or by sol–gel synthesis of inorganic nanostructures with subsequent polymer backfilling (Barbé *et al.*, 1997 ▸; Müller-Buschbaum *et al.*, 2017 ▸; Coakley *et al.*, 2005 ▸). For the latter case, mesoporous titania has been found to be a highly auspicious material in organic–inorganic bulk heterojunctions due to its stability (Zhang & Banfield, 1998 ▸), versatility (Su *et al.*, 2012 ▸; Rajeshwar *et al.*, 2001 ▸), properties as an electron conductor, as well as relative non-toxicity (Shi *et al.*, 2013 ▸), abundance and availability (Primo *et al.*, 2011 ▸). Many ways to synthesize titania films have been explored and methods have been refined to reliably produce titania films in various geometries, porosities, film thicknesses and crystal phases (Yusuf *et al.*, 2001 ▸; Tang *et al.*, 1994 ▸; Moret *et al.*, 2000 ▸; Liu *et al.*, 2005 ▸). However, good device performance is constrained by inefficient backfilling due to a lack of fundamental understanding of which factors influence backfilling efficiencies. Therefore, we investigate the backfilling of two different materials, namely the conjugated polymer poly[4,8-bis­(5-(2-ethyl­hexyl)­thio­phen-2-yl)benzo[1,2-*b*;4,5-*b*′]di­thio­phene-2,6-diyl-*alt*-(4-(2-ethyl­hexyl)-3-fluoro­thieno[3,4-*b*]thio­phene-)-2-carboxyl­ate-2-6-diyl)] (PTB7-Th) and the small molecule phenanthrene-fused tellurophene (PhenTe-BPinPh) (Hupf *et al.*, 2019 ▸). Both have the same volumetric mass density but differ very obviously in their size in solution [Fig. 1 ▸(*a*)].

PTB7-Th has attracted significant research attention as a stable low-bandgap organic semiconducting polymer. The recent exceptional performance achieved in organic photovoltaic (OPV) applications gives us reason to believe that PTB7-Th could be also successfully implemented in hybrid photovoltaic configurations (Meng *et al.*, 2018 ▸; Wan *et al.*, 2016 ▸; He, Xiao *et al.*, 2015 ▸). Although more chemically stable than the similar polymer PTB7, PTB7-Th also possesses a smaller band gap with a broader absorption wavelength range (Liao *et al.*, 2013 ▸; Bencheikh *et al.*, 2015 ▸). In addition, it has been shown that interaction with additives and ternary components in OPVs can greatly enhance device performance, thereby opening a large range of combination opportunities (Sun *et al.*, 2016 ▸).

This circumstance is particularly alluring with the dawn of the novel phenanthrene-fused tellurophene PhenTe-BPinPh [Fig. 1 ▸(*a*)] to act as a backfilling material. Being a heavy-element containing small molecule, it is expected to exhibit advantageous properties such as increased exciton diffusion length as well as improvements in conductivity owing to conjugated bonds comprising the molecular structure (He, Wiltshire *et al.*, 2015 ▸; Luppi *et al.*, 2019 ▸). In addition, heavy elements increase the probability of intersystem crossing (ISC) from an excited singlet state to an excited triplet state (Arif *et al.*, 2009 ▸; Gastilovich *et al.*, 2008 ▸). This directly combats one of the leading factors inhibiting organic and hybrid photovoltaic device performance, namely their limited exciton diffusion length (Forrest, 2005 ▸; Stranks *et al.*, 2013 ▸). The resulting longer excitonic lifetime directly translates into a greater diffusion length and ultimately fewer recombination losses.

Although much attention has been focused on the photovoltaic performance of PTB7-Th and the exciton lifetime-enhancing properties of heavy-element containing small molecules, reported work concerning the infiltration behavior of these materials into inorganic mesoporous structures is limited. As these materials appear promising for future hybrid photovoltaic configurations, their suitability for creating desirable interfaces is of special interest. In this study, we fabricate mesoporous titania films from a diblock copolymer templated sol–gel approach, which are then infiltrated with PTB7-Th and PhenTe-BPinPh, respectively. Both materials are proven to possess the same volumetric density as measured with X-ray reflectometry (XRR). Their respective backfilling efficiencies compared with unfilled titania films are investigated with time-of-flight grazing incidence small-angle neutron scattering (ToF-GISANS) (Müller-Buschbaum *et al.*, 2009 ▸). Neutron scattering delivers a high contrast that allows the detection of scattering signals from lighter atoms such as hydrogen, thereby providing a powerful tool in visualizing the materials used (Müller-Buschbaum, 2013 ▸). In the ToF mode, wavelength-resolved neutron scattering with a large probed *q*-range and high accuracy becomes possible due to statistical minimization of error (Müller-Buschbaum *et al.*, 2009 ▸). Our resulting morphological findings lay the groundwork for future research on the successful incorporation of PTB7-Th and PhenTe-BPinPh into hybrid photovoltaic devices and contribute a deeper understanding of influences on infiltration efficiency.

## 2. Experimental   

### 2.1. Sample preparation   

The synthesis of mesoporous titania films was based on an adapted sol–gel fabrication route described in the literature (Song *et al.*, 2017 ▸). More details can be found in the supporting information.

Infiltration of PTB7-Th and PhenTe-BPinPh into the mesoporous titania film was carried out according to a method inspired from the literature (Rawolle *et al.*, 2013 ▸). Prior to infiltration, the mesoporous titania thin films were submerged in the respective solvents (chloro­benzene for PTB7-Th and thf for PhenTe-BPinPh) for at least 45 min to remove air trapped inside the mesopores. For PTB7-Th infiltration, a solution (3.5 mg ml^−1^) was applied to the film surface (2 min soaking) and the excess solution was removed by spinning (600 rpm for 10 s, followed by 1500 rpm for 50 s). The solution deposition and spinning steps were repeated three times. PhenTe-BPinPh was infiltrated using a solution (10 mg ml^−1^) which was applied to the mesoporous titania thin film (1 min soaking) and spinned at 1000 rpm for 60 s. Note that different backfilling processing parameters were chosen in order to optimize the backfilling efficiency for the respective solvent/material system.

### 2.2. Scanning electron microscopy   

Scanning electron microscopy (SEM) images were taken on an FESEM Gemini NVision 40 workstation by Carl Zeiss Microscopy GmbH, controlled by the software SmartSEM, with an acceleration voltage of 5 kV and working distances from 3.3 to 4.6 mm. Samples were prepared for cross-section images by cooling in liquid nitro­gen and subsequent breaking. The cross-section images were taken under a tilt angle of 45° which was then corrected by software.

### 2.3. X-ray reflectometry   

X-ray reflectometry (XRR) measurements were conducted with monochromatic X-rays (Cu *K*α, λ = 1.54 Å) from a copper X-ray tube at a Bruker D8 Advance and a PANanalytical Empyrean X-ray diffractometer. The resulting reflectivity profiles were fitted using the Abeles matrix method implemented in the reflectivity analysis package *MOTOFIT* (Nelson, 2006 ▸) to obtain scattering length densities (SLDs), densities and film thicknesses. A solvent vapor annealing (SVA) technique was also explored as a possible method for film homogeneity enhancement.

### 2.4. Time-of-flight grazing incidence small-angle neutron scattering   

ToF-GISANS measurements were conducted at the REFSANS instrument of the Helmholtz Zentrum Geesthacht at the research neutron source Heinz Maier-Leibnitz (FRM II) in Garching, Germany (Moulin & Haese, 2015 ▸; Kampmann *et al.*, 2006 ▸), utilizing neutrons in the wavelength range 3.0–20.0 Å. The incident angle was fixed at 0.62° for all experiments, and effects due to gravity were corrected in the analysis and a detailed description of this correction can be found in the supporting information. The sample was mounted on top of a goniometer forming a fixed angle of 0.62° with respect to the horizon line. Due to gravity-induced wavelength-dependent neutron trajectories, real incident angles impinging on the sample range from 0.62 to 0.69° (see Fig. S7 of the supporting information) and the analysis was corrected for this effect. The arrival times and positions of scattered neutrons were detected on a (680 × 500) mm^2^ 2D multiwire ^3^He detector located at a fixed sample detector distance of 10.375 m. In order to receive sufficient statistics, a counting time of about 23 h per sample was chosen. A beam stop positioned in front of the detector was installed to shield off the direct beam and avoid oversaturation of the detector. The time-of-flight mode allowed binning of the recorded data into 20 wavelength slices with a respective resolution of Δλ/λ = 5.7% for analysis. Vertical and horizontal line cuts of resulting 2D detector images were conducted using the software tool *DPDAK* (Benecke *et al.*, 2014 ▸).

## 3. Results and discussion   

### 3.1. Scattering length density   

To be able to quantitatively analyze ToF-GISANS data obtained and make conclusions about the porosity and polymer infiltration efficiency, knowledge of the neutron scattering length densities of all used materials is paramount. Therefore, XRR measurements are conducted with thin films of pure PTB7-Th and PhenTe-BPinPh. Several samples are produced on different substrates and with varying deposition parameters to find the conditions which produce the most homogeneous films as confirmed with optical microscopy and XRR. Fig. 1 ▸ shows the XRR data obtained together with the fits to the data resulting from the Abeles matrix method. From the fits in Figs. 1 ▸(*b*) and 1 ▸(*c*), the X-ray SLD for PTB7-Th is determined to be SLD_*X*,PTB7-Th_ = 11.254 ± 0.108 × 10^−6^ Å^−2^, corresponding to a volumetric density of ρ_PTB7-Th_ = 1.241 ± 0.012 g cm^−3^ and a neutron SLD of SLD_*n*,PTB7-Th_ = 1.234 ± 0.012 × 10^−6^ Å^−2^. For the case of PhenTe-BPinPh, the fits in Figs. 1 ▸(*d*) and 1 ▸(*e*) yield an X-ray SLD of SLD_*X*,PhenTe-BPinPh_ = 10.554 ± 0.375 × 10^−6^ Å^−2^, corresponding to a volumetric density of ρ_PhenTe-BPinPh_ = ρ_PTB7-Th_ = 1.241 ± 0.044 g cm^−3^ and a neutron SLD of SLD_*n*,PhenTe-BPinPh_ = 1.619 ± 0.057 × 10^−6^ Å^−2^. Therefore, the same volumetric density of the custom-tailored PhenTe-BPinPh compared with PTB7-Th is found.

### 3.2. Film morphology   

**Figure 2:**
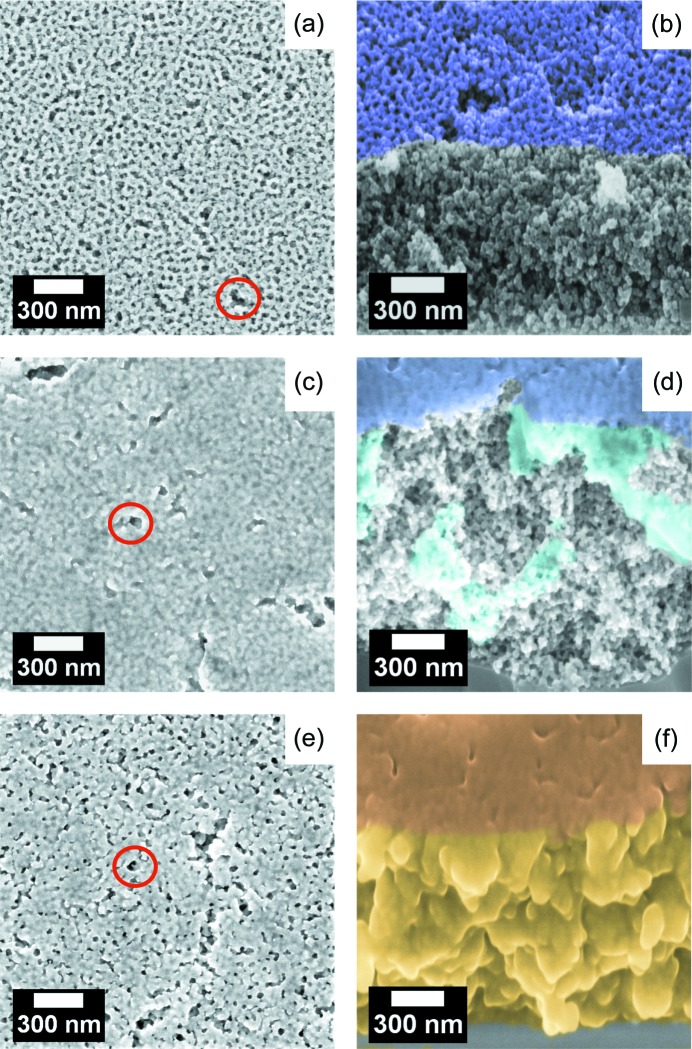
(*a*), (*c*) and (*e*) Surface SEM images and (*b*), (*d*) and (*f*) cross-section SEM images of mesoporous titania films (*a*) and (*b*) as-prepared and (*c*)–(*f*) backfilled. Defects are highlighted with red circles. (*b*) The top surface of the sample is purple with the cross-section kept in grayscale. (*d*) PTB7-Th overlayer represented in dark blue, with lighter blue showing the infiltrated polymer, and the as-prepared material kept in grayscale. (*f*) PhenTe-BPinPh backfilling, with the top surface shown in darker orange and backfilled cross-section in yellow.

Surface and cross-section SEM images of as-prepared and backfilled mesoporous titania thin films are shown in Fig. 2 ▸. Surface SEM of the as-prepared mesoporous titania network [Fig. 2 ▸(*a*)] shows homogeneously distributed pores with only a few defects. Cross-section SEM [Fig. 2 ▸(*b*)] reveals a mesoporous bulk network. In the case of titania thin film infiltration with PTB7-Th [Fig. 2 ▸(*c*)], near-surface pores appear to be efficiently filled. In addition, the surface of the mesoporous titania thin films is most likely completely covered by a polymer-capping layer. Study of the cross-section [Fig. 2 ▸(*d*)] reveals that PTB7-Th infiltration into deeper mesoporous layers is only partially achieved. Instead, most of the infiltration material is found to agglomerate in near-surface layers and a PTB7-Th-capping layer is observed. For the case of PhenTe-BPinPh-infiltrated titania thin films [Fig. 2 ▸(*e*)], foam-like structures appear with an increased diameter compared with as-prepared thin films [Fig. 2 ▸(*a*)]. A smaller number density of pores compared with as-prepared films is observed due to complete PhenTe-BPinPh backfilling of individual pores. In comparison with infiltration with PTB7-Th, no smearing-out of surface structures is detected, which indicates the absence of a PhenTe-BPinPh-capping layer. From cross-section SEM image analysis [Fig. 2 ▸(*f*)], a complete PhenTe-BPinPh filling of the mesoporous titania thin film is suggested, leading to a reduced visibility of the original foam-like titania framework. Within this scope, the observed structure almost appears as a single compact material phase instead of a mesoporous structure.

### 3.3. Porosity and infiltration efficiency   

**Figure 3:**
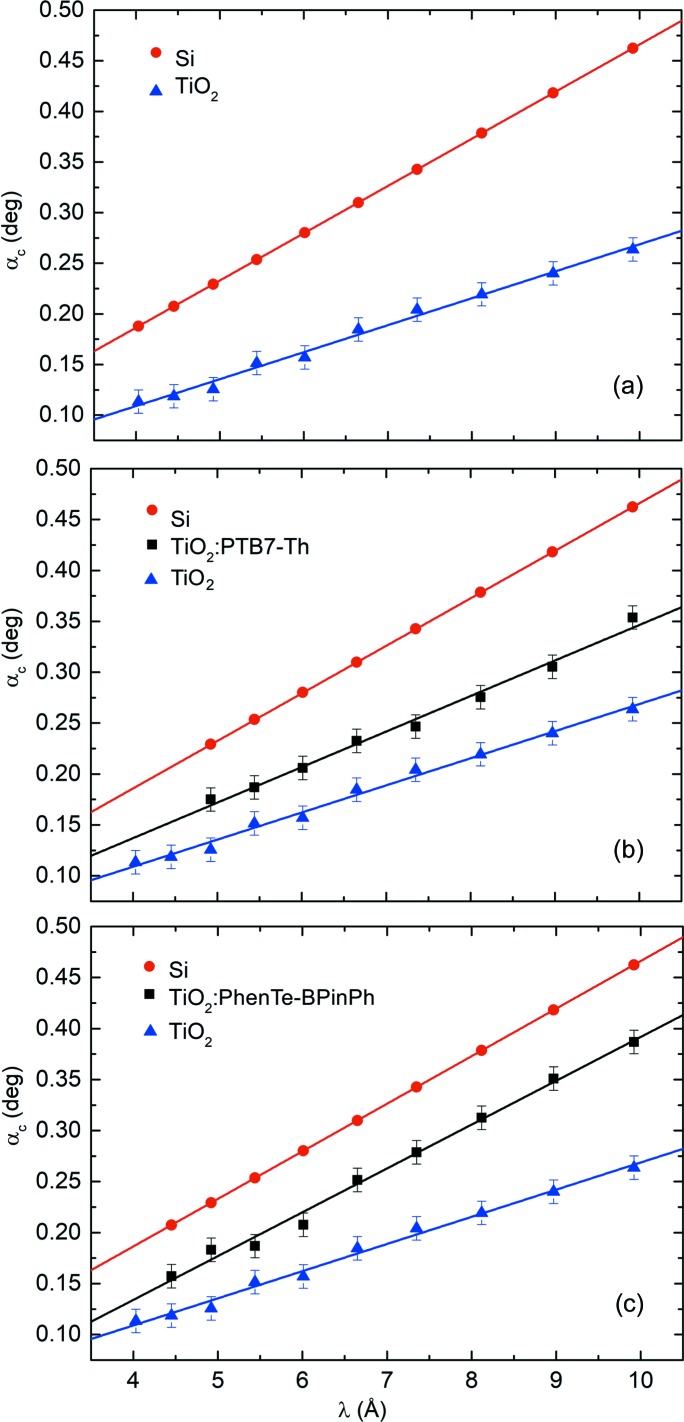
Linear regressions of material-specific critical angles obtained from vertical line cuts of ToF-GISANS data versus neutron wavelength for (*a*) as-prepared mesoporous titania, (*b*) mesoporous titania filled with PTB7-Th and (*c*) mesoporous titania filled with PhenTe-BPinPh. As indicated in the inset, different colors correspond to linear regressions related to the SLDs of Si (red) and TiO_2_ [TiO_2_: PTB7-Th (blue) and TiO_2_: PhenTe-BPinPh (black)] composite materials.

In order to gain quantitative information about the porosity of as-prepared mesoporous titania thin films and respective backfilling efficiencies of PTB7-Th and PhenTe-BPinPh, vertical line cuts (along *q_z_* at *q_y_* = 0) of 2D ToF-GISANS data are performed. A wavelength range from 4.0 to 9.9 Å is chosen and neutrons in this specific range penetrate the whole film. Hence, bulk material sensitivity is ensured. In Fig. 3 ▸, extracted peak positions corresponding to the material-specific critical angles (Yoneda peaks; Yoneda, 1963 ▸) are plotted against the respective neutron wavelength band. More information on the determination of Yoneda peak positions and the fits for determining the Yoneda peak positions in respective vertical line cuts is provided in the supporting information. Linear regression (solid lines) is carried out to determine the SLDs of as-prepared mesoporous TiO_2_ and of backfilled composite TiO_2_: PTB7-Th and TiO_2_: PhenTe-BPinPh thin films. For as-prepared mesoporous titania, a neutron SLD of SLD_TiO_2__ = 6.79 ± 0.43 × 10^−7^ Å^−2^ is determined. Comparison with compact titania yields a porosity of Φ = 71.1 ± 1.8%. For backfilled composite films, linear regressions for respective SLD extraction are depicted in Figs. 3 ▸(*b*) and 3(*c*). Accordingly, SLDs of SLD_PTB7-Th_ = 1.16 ± 0.10 × 10^−6^ Å^−2^ and SLD_PhenTe-BPinPh_ = 1.77 ± 0.12 × 10^−6^ Å^−2^ are calculated, resulting in backfilling efficiencies of ξ_PTB7-Th_ = 39.4 ± 8.5% and ξ_PhenTe-BPinPh_ = 67.1 ± 7.7%, respectively.

Note that the values for porosity and backfilling efficiency are not obtained from an infinitely sharp Yoneda peak. A distribution of local porosities and backfilling efficiencies in the respective sample causes broadening of the peak (Rawolle *et al.*, 2013 ▸). In particular, differences between the bulk and the surface as well as fluctuations in the horizontal plane are possible. The indicated porosity is an average value representing the entire film. SEM images confirm this hypothesis of lateral and vertical backfilling differences; a prime example is the case of PTB7-Th infiltration in Figs. 2 ▸(*c*) and 2(*d*), where certain areas appear to be backfilled more than others. In contrast, the porous structure of the as-prepared titania films [Fig. 2 ▸(*a*) and 2(*b*)] and the infiltrated morphology with small molecules [Figs. 2 ▸(*e*) and 2 ▸(*f*)] appear homogeneous in all directions. Therefore, the extracted porosity of as-prepared mesoporous titania and the extracted backfilling efficiency for PhenTe-BPinPh represent the whole film morphology.

### 3.4. Inner-film morphology   

**Figure 4:**
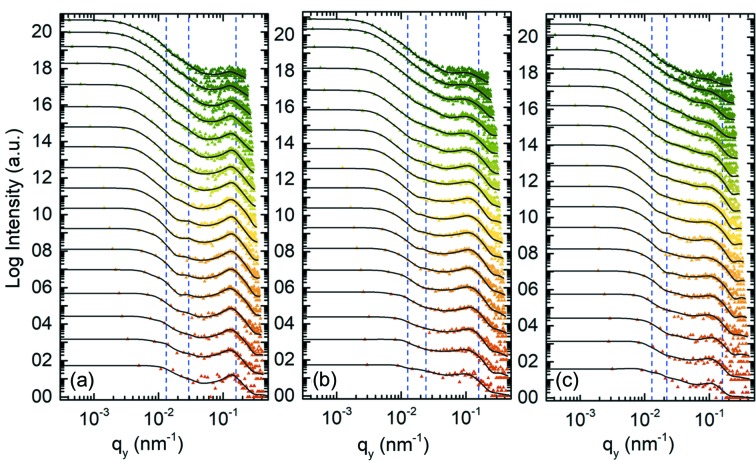
Horizontal line cuts for (*a*) the as-prepared mesoporous titania film, (*b*) PTB7-Th infiltrated titania and (*c*) PhenTe-BPinPh infiltrated titania. Solid lines represent the best model obtained for the data and dashed lines indicate the peak position of modeled structure factors. The curves are shifted along the intensity axis for clarity. Neutron wavelength bands increase from bottom to top (3.3–18.1 Å).

In the following, information about lateral structures (parallel to the substrate surface) inside the respective thin films is extracted. Horizontal line cuts (in the *q_y_* direction) of 2D ToF-GISANS data are performed for different wavelength bands by averaging over 11 pixels in *q_z_* around the position of maximum lateral scattering intensity. The resulting cuts for each wavelength band and sample can be found in Fig. 4 ▸. Within this scope, a prominent feature between high *q*-values from 0.07 to 0.2 nm^−1^ is clearly visible for all samples. The *q*-range of this feature corresponds to the length scales of the mesoscale structure observed via SEM and is therefore the main object of scrutiny. A second feature at lower *q*-values between 0.02 and 0.04 nm^−1^ and a third feature between *q*-values 0.009 and 0.018 nm^−1^, somewhat hidden in the shoulder, can also be found. Quantitative information can be extracted by modeling of the horizontal line cut data using the Local Monodisperse Approximation (LMA) and Effective Interface Approximation (EIA) of cylindrically shaped structures distributed on a 1D paracrystal lattice in combination with the Distorted Wave Born Approximation (DWBA) (Vineyard, 1982 ▸; Hosemann *et al.*, 1981 ▸; Müller-Buschbaum, 2009 ▸). Three form factors (FFs) corresponding to the cylinder radii and three structure factors (SFs) describing the center-to-center distances of the repeating structures are implemented for data modeling for all wavelength bands (Li *et al.*, 2016 ▸; Renaud *et al.*, 2009 ▸; Lazzari *et al.*, 2007 ▸). For as-prepared mesoporous titania [Fig. 4 ▸(*a*)], the first feature in the *q*-value range 0.07–0.2 nm^−1^ is very strongly pronounced. With PTB7-Th [Fig. 4 ▸(*b*)] and PhenTe-BPinPh [Fig. 4 ▸(*c*)] infiltration, this feature gradually loses definition. This smearing indicates deposition of the backfilled material into the titania matrix, or, in other words, partially filled pores reducing the uniformity of the overall observed average structure. A detailed view of the first feature can be found in Fig. S12.

For as-prepared mesoporous titania, FF_1,as-prepared_ = 11.5 ± 0.1 nm is extracted via data modeling for the smallest form factor. Furthermore, infiltration with the macromolecule PTB7-Th yields an increased smallest form factor FF_1,PTB7-Th_ = 13.6 ± 0.1 nm. The largest smallest form factor FF_1,PhenTe-BPinPh_ = 15.9 ± 0.2 nm is obtained for infiltration with the small molecule PhenTe-BPinPh. Respective structure factors remain almost constant at values of SF_1,as-prepared_ = 39.6 ± 0.1 nm, SF_1,PTB7-Th_ = 39.6 ± 0.2 nm and SF_1,PhenTe-BPinPh_ = 39.5 ± 0.2 nm. Modeling thereby suggests that the structure positions of the rigid inorganic framework and respective center-to-center distances are not affected by infiltration. In contrast, the average structure size is found to change depending on the infiltrated material.

**Figure 5:**
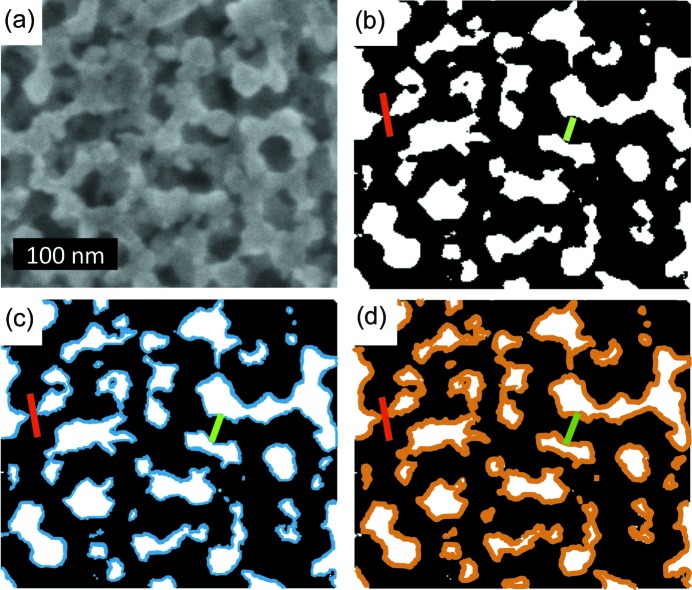
Schematic representation of the material infiltration model into a mesoporous titania matrix. (*a*) Grayscale SEM image of as-prepared mesoporous titania and (*b*) binarized SEM with structure size (2 × FF_1_, green bar) and distance (SF_1_, red bar) as extracted from ToF-GISANS modeling for as-prepared mesoporous titania. SEM images with the respectively growing structure sizes (2 × FF_1_, green bars) and constant distances (SF_1_, red bars) upon pore infiltration with (*c*) PTB7-Th represented in blue and (*d*) PhenTe-BPinPh represented in orange. The simulated blue and orange representations of PTB7-Th and PhenTe-BPinPh are added for visualization.

Extracted information from SEM and ToF-GISANS is used to develop a model describing the backfilling processes. For this purpose, an unaltered SEM image of an as-prepared titania film is shown in Fig. 5 ▸(*a*). Binarization using Yen’s thresholding method (Yen *et al.*, 1995 ▸; Sezgin & Sankur, 2004 ▸) is applied with the software *ImageJ* [Fig. 5 ▸(*b*)]. Therefore, a simplified visualization representing titania in black and pores in white is obtained, which is used in the following to explain the modeling results extracted from ToF-GISANS data. The green bar in Fig. 5 ▸(*b*) represents the cylinder diameter (FF_1,as-prepared_ × 2) and the red bar represents the center-to-center (SF_1,as-prepared_) distance between two cylinders. In the model developed, backfilling this mesoporous matrix with PTB7-Th [Fig. 5 ▸(*c*)] or PhenTe-BPinPh [Fig. 5 ▸(*d*)] leads to the respective material being deposited on the pore walls (blue and orange) and a consequential increase of the cylinder diameter. The center-to-center distance remains unaffected by the additional material and is constant. Note that in the model presented here, precise values, as extracted from ToF-GISANS data modeling, are transferred into the SEM representation true to scale. Therefore, SEM and ToF-GISANS results are in good agreement.

Through the second feature (in between *q*-values from 0.02 to 0.04 nm^−1^), a form factor of FF_2,as-prepared_ = 66.8 ± 1.1 nm is extracted for as-prepared mesoporous titania. This form factor decreases to FF_2,PTB7-Th_ = 62.9 ± 1.0 nm and FF_2,PhenTe-BPinPh_ = 47.6 ± 1.1 nm for PTB7-Th and PhenTe-BPinPh infiltration, respectively. In contrast, the corresponding structure factor is found to increase from SF_2,as-prepared_ = 213 ± 3 nm for the as-prepared case to SF_2,PTB7-Th_ = 259 ± 3 nm and to SF_2,PhenTe-BPinPh_ = 286 ± 9 nm for PTB7-Th and PhenTe-BPinPh infiltration, respectively. Defects with matching length scales (marked by red circles in Fig. 2 ▸) are assumed to be the origin of this second feature. Note that according to Babinet’s principle, the scattering of a body is equal to that from a hole of the same size and shape (Guenther, 2015 ▸). Therefore, solid titania structures as well as holes can be seen as the origin of the resulting scattering pattern. When the above-mentioned defects are backfilled with material, the size of the holes is reduced, which is reflected in the trend of decreasing form factors FF_2_ with improving backfilling efficiency. Similarly, with increasing backfilling efficiency, the likelihood of complete pore filling is also increased. A simplified case of a single pore in between two partially filled pores being completely backfilled would translate to a doubling of the local structure factor in the applied model. Considering the entireness of the thin film, increased occurrence of complete pore filling of individual pores leads to an increase of the average structure factor. This exact behavior is seen as the origin of the above-described increase of structure factor SF_2_ with increasing backfilling efficiency.

Modeling related to the third feature (in between *q*-values from 0.009 to 0.018 nm^−1^) is seen to reflect large-scale aggregations and local fluctuations of material density. These fluctuations can result from domains of slightly tighter packed titania granules in the case of as-prepared mesoporous samples and domains of locally higher backfilling efficiency in the case of infiltrated samples. No trend correlating to backfilling efficiency is observed. In detail, form factors of FF_3,as-prepared_ = 173 ± 2 nm, FF_3,PTB7-Th_ = 157 ± 2 nm and FF_3,PhenTe-BPinPh_ = 164 ± 3 nm for as-prepared PTB7-Th backfilling and PhenTe-BPinPh backfilling are extracted, respectively. Related structure factors are SF_3,as-prepared_ = 479 ± 12 nm, SF_3,PTB7-Th_ = 499 ± 3 nm and SF_3,PhenTe-BPinPh_ = 488 ± 8 nm.

During the backfilling process, many factors influence how well the respective materials can infiltrate the mesoporous titania network. Key influences such as solution viscosity, material mobility, temperature, soaking time, concentration and molecular weight determine the interaction between solution and titania network and therefore how well material is deposited (Coakley *et al.*, 2003 ▸; Frank *et al.*, 1996 ▸; Bartholomew & Heeger, 2005 ▸). In our case, even though several of these factors such as soaking time and concentration favor better backfilling efficiencies for PTB7-Th and, in spite of the same volumetric density of both materials, PhenTe-BPinPh had a 1.7 times higher backfilling efficiency. Therefore, higher infiltration efficiency of tellurophene compared with PTB7-Th is assumed to be primarily caused by the difference in respective molecule sizes. In contrast to small molecules, polymers have long backbones with pendant groups which can facilitate entanglement of the chains and formation of aggregates hindering good infiltration (Nguyen *et al.*, 1999 ▸; Bencheikh *et al.*, 2015 ▸). Therefore, it is necessary to find conditions in which the polymer chains retain their mobility and refrain from forming larger structures that would be less likely to infiltrate pores.

## 4. Conclusions   

Mesoporous titania films synthesized using a sol–gel diblock copolymer templating method are subsequently backfilled with the macromolecule PTB7-Th and the custom-made small molecule PhenTe-BPinPh. XRR measurements of pure PTB7-Th and PhenTe-BPinPh confirm the same volumetric density of both materials while having very different molecular sizes. As the two materials are of different material classes, namely a polymer and a small molecule, many structural properties differ. However, the mass densities are identical, so this property can be excluded as an influencing factor on the backfilling efficiency. The infiltration is examined by comparing infiltrated titania films with as-prepared mesoporous titania films via ToF-GISANS. A porosity of 71.1% is found for the as-prepared mesoporous titania film, whereas infiltration efficiencies of 39.4% for PTB7-Th and 67.1% for PhenTe-BPinPh are achieved. Structures on three different length scales are found through data modeling. Hence, sizes attributed to the mesoscopic foam-like structure are seen to increase with enhanced backfilling efficiency. In contrast, the distance between structures remains stable due to the inorganic robust framework in the form of mesoporous titania. Complementary SEM images disclose the formation of a sealing overlayer during PTB7-Th backfilling, while PhenTe-BPinPh homogeneously penetrates the whole film volume.

Thus, in the present study, structural changes induced through infiltration with two different and highly promising materials for photovoltaic applications into mesoporous titania are observed. Different backfilling efficiencies are detected despite the two materials having the same volumetric density. Within this scope, the good infiltration efficiency for PhenTe-BPinPh is mainly ascribed to a smaller molecule size compared with the macromolecule PTB7-Th. From the present study, future optimization processes of backfilling in hybrid photovoltaic approaches can be assumed to be facilitated by providing the knowledge that the volumetric density is not a key influence to be considered for infiltration efficiency.

## 5. Related literature   

The following references are cited in the supporting information: Greenwood & Earnshaw (1988 ▸); Attwood (1999 ▸); Chantler *et al.* (2005 ▸); Fu *et al.* (2018 ▸); Kaune *et al.* (2010 ▸); Müller-Buschbaum (2003 ▸); Rauch & Waschkowski (2003 ▸); Roe (2000 ▸).

## Supplementary Material

Supporting information file. DOI: 10.1107/S2052252520000913/ct5013sup1.pdf
