# Supplementary material for: Comparing the backfilling of mesoporous titania thin films with hole conductors of different sizes sharing the same mass density
Source: IUCrJ. 2020 Feb 12;7(Pt 2):268–75. doi: 10.1107/S2052252520000913 (PMC7055378; doi:10.1107/S2052252520000913)
Supplement: Supplementary file 1 [file m-07-00268-sup1.pdf]

IUCrJ

Volume 7 (2020)

Supporting information for article:

Comparing the backfilling of mesoporous titania thin films with hole conductors of different size sharing the same mass density

Raphael S. Märkl, Nuri Hohn, Emanuel Hupf, Lorenz Bießmann, Volker Körstgens, Lucas P. Kreuzer, Gaetano Mangiapia, Matthias Pomm, Armin Kriele, Eric Rivard and Peter Müller-Buschbaum

S1. Preparation of pure PTB7-Th and PhenTe-BPinPh films

PTB7-Th purchased from 1-Material was dissolved in chlorobenzene (3.5 mg/mL). The solution was stirred at 60 °C and 600 rpm for 1.5 h. Subsequently, the solution was spin-coated (Süss MicroTec Delta6 RC) onto respective glass and silicon substrates. Spin coating parameters were changed to achieve optimized homogeneity. For glass substrates, 1000 rpm for 60 s and subsequent 1500 rpm for 60 s were used, while for silicon substrates, 1000 rpm for 60 s were used. Parameters were based on successful optimization of processing conditions.

PhenTe-BPinPh was synthesized in the Rivard Group at the University of Alberta and was dissolved in THF (10 mg/mL, purity > 99.8%) (Hupf *et al.*, 2019). The solution was stirred at 500 rpm and room temperature for 1.5 h. A solvent vapor annealing (SVA) technique was also applied for some samples to increase film homogeneity by depositing 1 mL of THF on the spin coater sample stage and placing a glass beaker (500 mL, Duran) over the stage after the **PhenTe-BPinPh** solution was deposited on the substrate. The sample then spun at 1000 rpm for 10 minutes. Parameters were based on successful optimization of processing conditions. Subsequently, solvent vapor annealing was continued for further 15 min.

S2. Titania sol-gel synthesis route (Song *et al.*, 2017)

100 mg of the amphiphilic diblock copolymer polystyrene-block-polyethylene oxide (PS-b-PEO, Polymer Source. Inc.) with a molecular weight distribution of M_n(PS) = 63 kg mol^{-1}, M_n(PEO) = 26 kg mol^{-1} were used as a templating agent. An azeotrope mixture of 3.35 mL toluene (purity > 99.8%) and 1.342 mL 1-butanol (purity > 99.8%) was successively added and stirred for 10 minutes. Subsequently, 369 µL of titanium (IV) isopropoxide (TTIP, purity > 97%) were added and the solution was stirred for 30 min. Thereafter, 121 µL HCl (6M, diluted from 37% / 12M) were added dropwise to the solution. The solution was stirred for further 20 h at 1000 rpm under ambient conditions. All solvents were purchased from Carl Roth and used without further processing.

The obtained solution was deposited onto acid cleaned (Müller-Buschbaum, 2003) silicon wafers pre-treated with oxygen plasma (Diener electronic GmbH & Co. KG, Nano) immediately before deposition. The solution was spin-coated at 1000 rpm for 10 s, after which the sample was thermally annealed at 100 °C for 5 min, then 200 °C for 5 min, and again 100 °C for 5 min on hot plates (IKA RCT Basic). To achieve a sufficient film thickness, the spin-coating and annealing steps were repeated 4 times. Finally, the samples were calcined at 300 °C for 30 min with a heating rate of 3 °C/min, followed by 500 °C for 2 h with a heating rate of 5 °C/min in a tube furnace (Gero

Hochtemperaturöfen GmbH) to remove the polymer template and to induce anatase-type crystallization.

S3. SLD profiles from XRR measurements

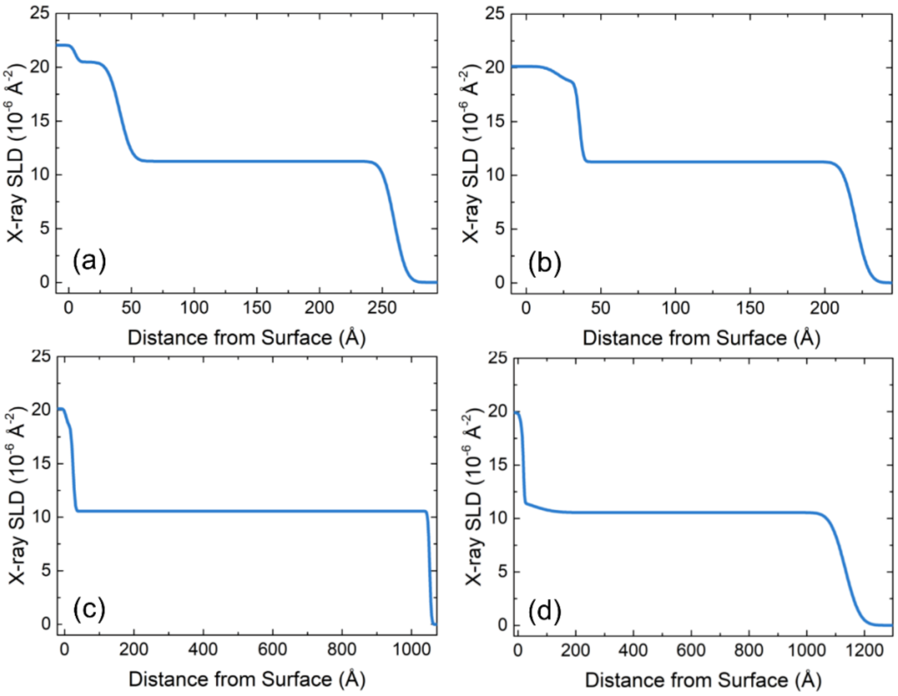

Figure S1 Profiles of X-ray scattering length density as function of the distance from the substrate surface as extracted from XRR measurements; (a) **PTB7-Th** spin-coated at 1000 rpm on glass, (b) **PTB7-Th** spin-coated at 1500 rpm on silicon, (c) **PhenTe-BPinPh** spin-coated at 1000 rpm on silicon with subsequent solvent vapor annealing (SVA) and (d) without SVA.

S4. Footprint correction for XRR measurements with the *Empyrean* (PANanalytical) X-ray diffractometer

The footprint correction corrects the intensity (Figure S2) for very small angles in the case that the X-ray beam width is larger than the sample length as follows

$$I_c = I_1 \frac{L_c}{L_1} = I_1 \frac{w}{sin\alpha\, L_1}$$

where I_c is the corrected intensity, I_1 is the uncorrected intensity, L_c is the corrected sample length in beam direction, L_1 is the real uncorrected sample length, w is the beam width in beam direction, and α is the incident angle. With the geometric relation

$$sin\alpha = \frac{h}{L_1} = \frac{w}{L_c}$$

where h is the fraction of w illuminating the sample, the footprint can be corrected with knowledge of the fixed beam width $w = 0.08$ mm which is determined by the used slit and the sample length of $L_1 = 20$ mm. The correction is done for small angles where $I_1 < I_c$.

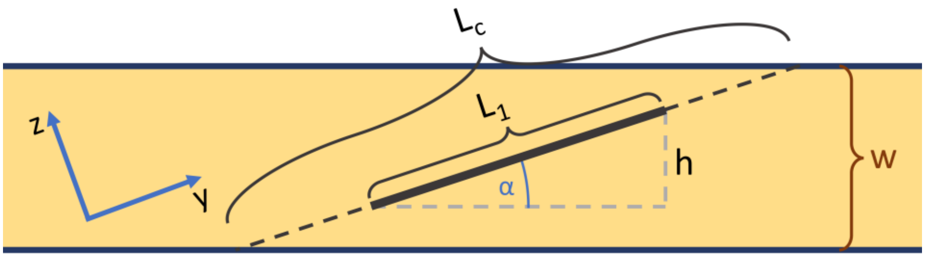

Figure S2 Geometrical depiction of parameters used for XRR footprint correction. Incident X-ray beam with width w represented in orange, impinging under an angle α on the sample with length L_1 represented in black. With the beam fraction h illuminating the sample, the corrected beam length L_c can be calculated.

S5. Calculation of density and neutron scattering length density

The Abeles matrix method implemented in Motofit was applied to XRR data and an X-ray SLD was obtained. From this, the material volumetric density and the neutron SLD was calculated. In good approximation the SLD is defined as (Roe, 2000):

$$SLD = \frac{2\pi\delta}{\lambda^2}$$

and the refractive index for X-rays is defined as (Attwood, 1999)

$$n = 1 - \delta + i\beta$$

with $\delta = \frac{n_a r_e \lambda^2}{2\pi} f_1$ and $\beta = \frac{n_a r_e \lambda^2}{2\pi} f_2$ for one atom,

where r_e is the classical electron radius, $n_a = \frac{\rho N_a}{M_a}$ is the number density, f_1 and f_2 are atomic scattering factors, ρ is the volumetric density, N_a is the Avogadro constant and M_a is the molar mass (g/mol). The scattering length for X-rays is defined as $b_i = r_e f_1$.

With the relation(Roe, 2000) $\theta_c = \sqrt{2\delta}$ and plugging the definition of δ into the equation for θ_c, using the definition for n_a, and solving for ρ yields:

$$\rho = \frac{{\theta_c}^2 \pi \sum_i^N c_i M_i}{N_a r_e \lambda^2 \sum_i^N c_i f_{1i}}$$

$$\rho = \frac{SLD \sum_i^N c_i M_i}{N_a r_e \sum_i^N c_i f_{1i}}$$

The form factors for single atoms were obtained from literature and the total form factors for **PTB7-Th** and **PhenTe-BPinPh** were calculated as follows (Chantler *et al.*, 2005):

PTB7-Th ($C_{49}H_{57}FO_2S_6$): $f_{tot} = 476.59$

PhenTe-BPinPh ($C_{28}H_{25}BO_2Te$): $f_{tot} = 266.63$

The scattering lengths b_i were different for neutrons than for X-rays and could also be obtained from literature (Rauch & Waschkowski, 2003). The relation between volumetric density and SLD was used to extract the neutron SLD by plugging in neutron scattering lengths b_i and the calculated volumetric density.

S6. Exemplary 2D ToF-GISANS image with respective features

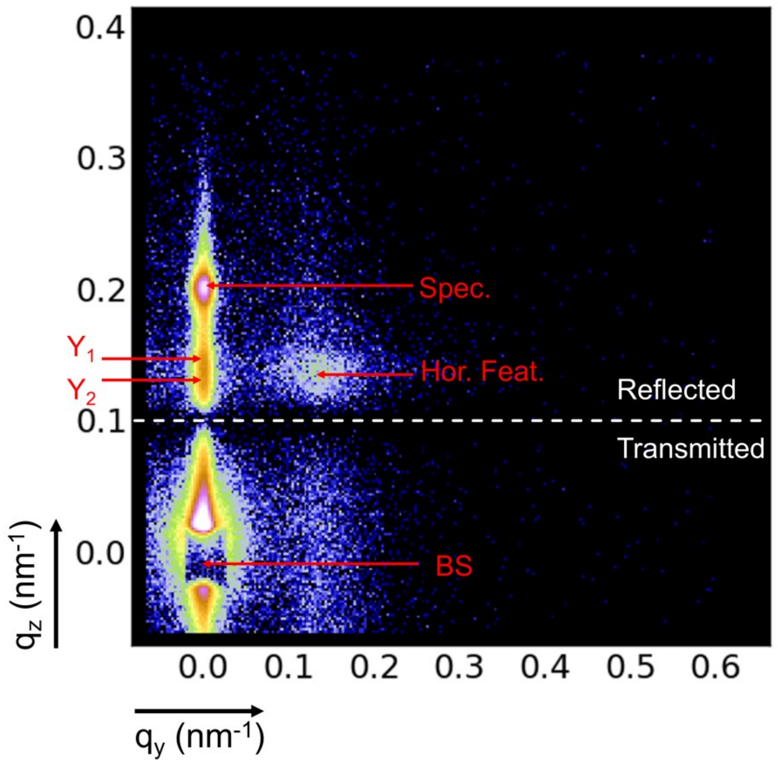

Figure S3 Exemplary 2D ToF-GISANS image divided into the upper part showing reflection and the lower part showing transmission. The specular beam (Spec.) is clearly visible and separated from the two Yoneda Peaks (Y1, Y2). Around the maximum lateral scattering intensity, the horizontal feature (Hor. Feat.) can be seen, from which horizontal structural information is gained. In the transmitted region, the square shaped beam stop (BS) partially blocks the direct beam.

S7. Selection of 2D ToF-GISANS images

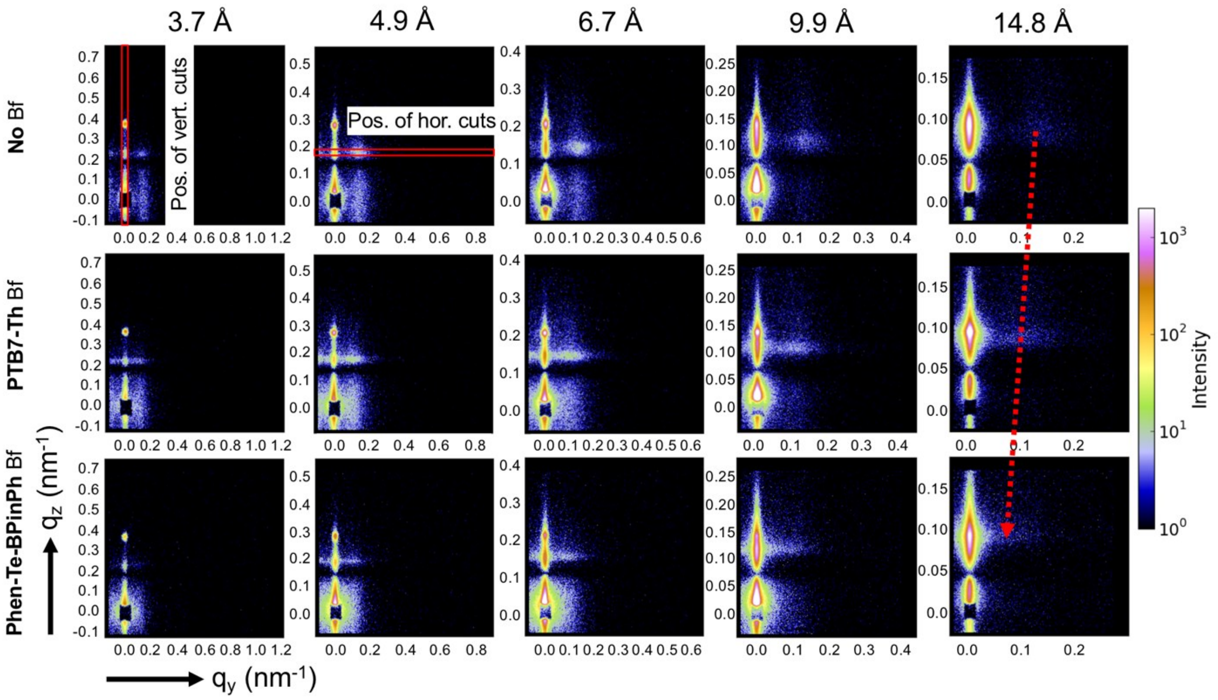

Figure S4 2D ToF-GISANS data for as-prepared, PTB7-Th infiltrated, and PhenTe-BPinPh infiltrated mesoporous titania at selected wavelength bands with positions of an exemplary vertical cut and horizontal cut (marked by red lines). A shift of the most prominent horizontal feature towards smaller q_y values can be observed for different backfilling materials (exemplarily marked as red dotted line).

S8. Schematic representation of backfilling

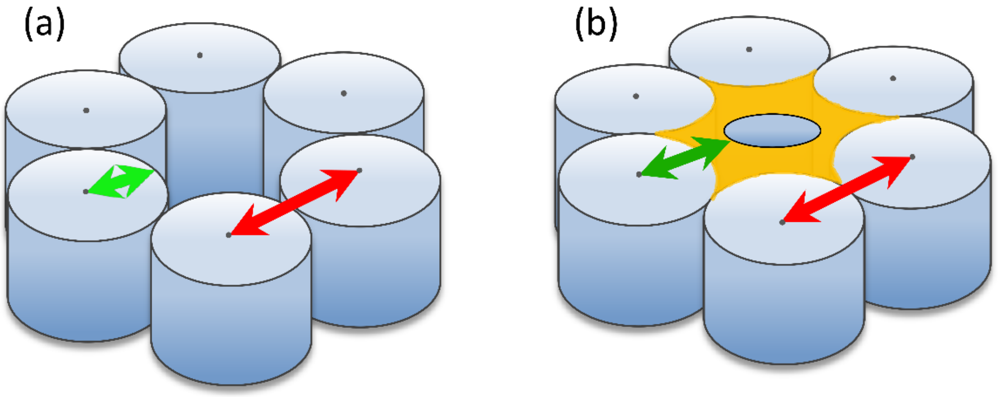

Figure S5 Schematic representation of the behavior of structure diameter (green arrows) and structure center-to-center distance (red arrows) as extracted from horizontal line cuts through

modeling. For as-prepared, mesoporous titania, the structure diameter is determined by cylindrical titania structures, arranged such that porous areas are formed and thereby defining the structure center-to-center distance. When material is infiltrated and adheres to pore walls, the average cylinder size grows, while the center-to-center distance remains constant.

S9. Gravitational correction in ToF-GISANS measurements

In GISANS and NR measurements, the nominal incident angle α_{gon} is usually determined by the slope of the sample surface (goniometer plane), while the incident beam is collimated and directed along the horizon. Here, we used $\alpha_{gon} = 0.62°$. However, (*i*) since REFSANS has a horizontal scattering geometry; (*ii*) because of the ToF-mode and of the wide wavelength range used; (*iii*) due to the vertical fall of neutrons caused by the presence of the gravitational field, the nominal value α_{gon} does not correspond to the real incident angle of the neutrons, α_{λ}, which will actually depend on the wavelength. The determination of the real incident angle is, of course, very important because ignoring it would result in a significant systematic error in the calculation of the q-values.

Figure S6 shows a schematic view of the scattering process in which the motion of neutrons with a certain wavelength λ is analyzed. The frame delimited by the dotted line on the right side represents a zoom of the scattering process occurring at the sample position. Starting from the last slit, the incident beam is directed towards the sample forming an incident angle of α_{λ} which is larger than the nominal angle α_{gon} because of the parabolic "trajectory" indicated in blue. In the absence of the gravitational field, the neutron motion would be a straight line, which is indicated in black. The transmitted beam continues its travel and reaches the detector plane at the position P_{λ}^{0}, which is lower than the hypothetical position of an undisturbed beam, indicated with T^{0}.

To correctly evaluate the real incident angle, it is necessary to apply a robust method, which is independent of the instrumental settings. What is done is to divide the neutrons recorded by the detector in wavelength slices each of which has a predefined width, which can be selected after the experimental investigation, since all the events are recorded in list-mode. For each slice, a ROI containing the specular signal on the detector is selected: This can be easily done though a vertical line cut performed along the horizontal position corresponding to the incident beam. The specular signal is easily detectable because it usually corresponds to the most intense one and even when this is not the case, it may be detected because it must be located around the nominal value α_{gon}, at least for the shortest wavelengths (with least gravitational effect). The vertical position (in pixels) P_{λ} of the specular signal is detected for each slice, fitting a Gaussian to the experimental data belonging to the appropriate chosen ROI. In figure S6, the trajectory of the specular reflected beam is also indicated, along with the hypothetical trajectory of the beam not experiencing the presence of the gravitational

field, which reaches the detector at the position T. A similar procedure is applied to determine P_λ^0: In this case the sample and the beam-stop are removed and the incident beam is directly measured on the detector, eventually using an attenuator to avoid saturating or damaging the detector.

As mentioned, during the travel from the sample position to the detector, both the transmitted and the specular beams have dropped because of the gravitational field: The essential point of the method adopted is based on the fact that their time-of flight is practically the same and, consequently, the vertical shift is the same. In fact, if D is the sample-detector distance, the time-of-flight of the direct beam, tof_{DB} is

$$tof_{DB} = \frac{D}{v_\lambda}$$

where v_λ is the scalar velocity of the neutrons of wavelength λ. It is important to note that the time of flight is independent of the gravitational acceleration g, provided that the evaluation of the distances and velocities occurs in the horizontal plane, where g has a null component. For the specular beam, a similar procedure gives the time-of-flight tof_R

$$tof_R = \frac{D}{v_\lambda \cos\alpha_\lambda}$$

The vertical shifts Δz_{DB} and Δz_R will be then

$$\Delta z_{DB} = g\frac{(tof_{DB})^2}{2} = g\frac{D^2}{2v_\lambda^2}$$

$$\Delta z_R = g\frac{(tof_R)^2}{2} = g\frac{D^2}{2v_\lambda^2\,(\cos\alpha_\lambda)^2}$$

For a typical value of α_{gon}, even for neutrons with a wavelength of 20 Å, the difference between Δz_{DB} and Δz_R over a distance of ≅ 10 m is of the order of few micrometers. Thus, it is negligible with respect to the detector pixel sizes (some millimeters).

Provided that the vertical shift is identical for both the direct and the specular beam, the real incident angle α_λ is readily obtained as

$$\frac{|P_\lambda - P_\lambda^0|s_z}{D} = \tan\alpha_\lambda$$

where s_z is the vertical pixel size, which converts the distance measured in pixels in a real value. The presence of the absolute value suppresses the necessity to know the orientation of the vertical axis.

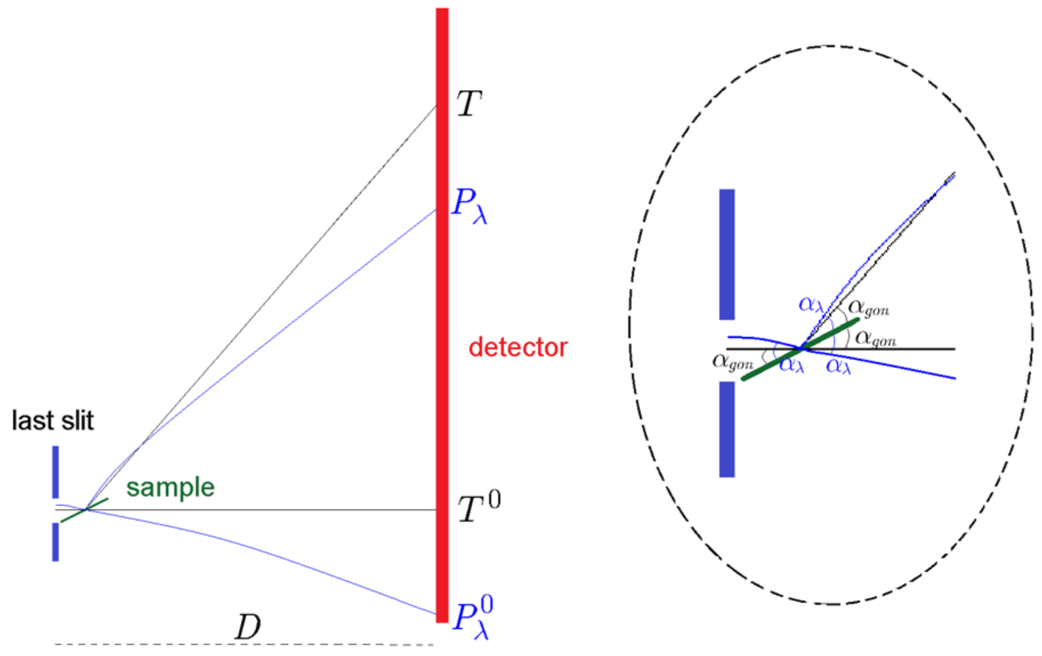

Figure S6 Schematic view of the scattering process during ToF-GISANS measurements of neutrons with a certain wavelength λ. The dotted circle on the right side represents a detailed view of the scattering process occurring at the sample position. Starting from the last slit on the left, the incident beam impinges on the sample, thereby forming an incident angle $\alpha_\lambda$which is larger than the nominal angle α_{gon} due to the parabolic "trajectory" indicated in blue. Without the gravitational field, the neutron trajectory would be a straight line, indicated in black. The transmitted beam continues its path and reaches the detector plane at the position P_λ^0, which is lower than the hypothetical position of an undisturbed beam, indicated with T^0. P_λ denotes the vertical position of the specular signal and T the hypothetical position of an undisturbed beam.

S10. ToF-GISANS real incidence angle and penetration depth

The wavelength dependent penetration depth of neutrons is determined by the ratio of incident angle and critical angle. Neutrons either deeply penetrate the film when the incident angle is larger than the critical angle, or are surface sensitive when the incident angle is smaller than the critical angle (Müller-Buschbaum, 2013). As shown in Figure S7, the real incident angles in the ToF-GISANS experiments ranged from 0.62° to 0.68° and thus were always larger than the critical angles of our investigated materials. Therefore, it can be concluded that all shown measurements are bulk sensitive.

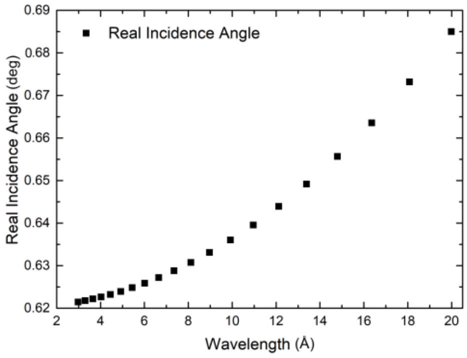

Figure S7 Wavelength dependent real incident angles of the ToF-GISANS measurements.

S11. Vertical line cuts of 2D ToF-GISANS images

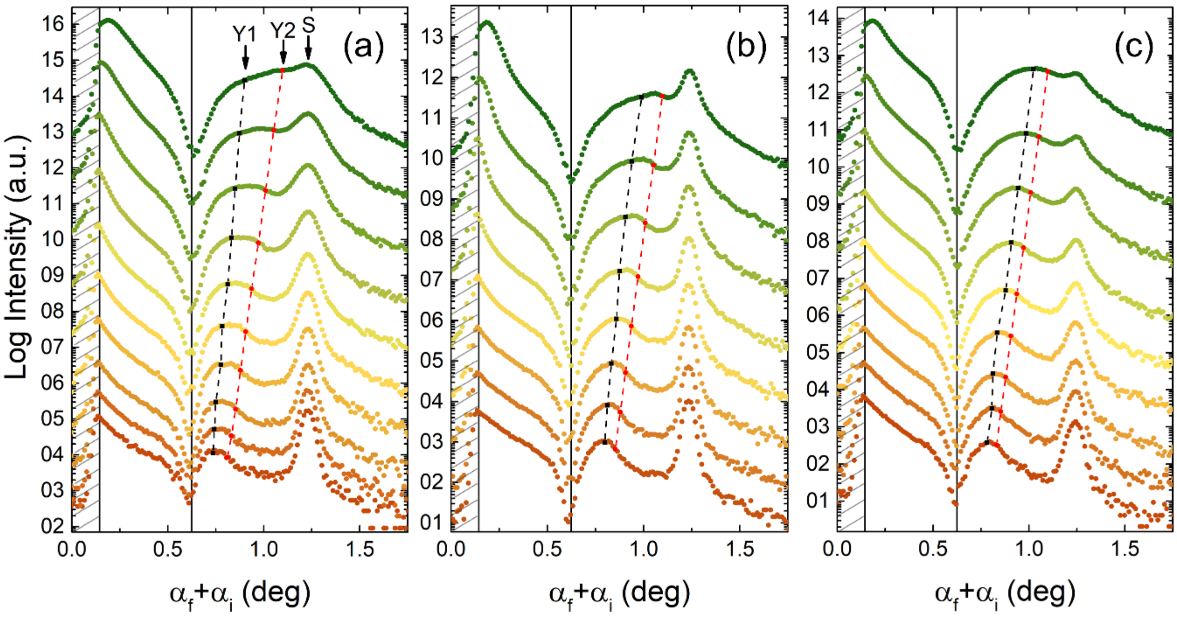

Figure S8 Intensities of vertical line cuts performed at $q_y = 0$ as an average over 15 pixels in q_y vs the scattering angle (α_f+α_i) for the titania film (a) as-prepared, (b) infiltrated with **PTB7-Th**, and (c) infiltrated with **PhenTe-BPinPh**. The cuts are stacked vertically along the intensity axis with neutron wavelength bands increasing from bottom to top for as-prepared titania (4.0 Å – 9.9 Å), for titania filled with **PTB7-Th** (4.9 Å – 9.9 Å) and for titania filled with **PhenTe-BPinPh** (4.5 Å – 9.9 Å). The black vertical line denotes the sample horizon and the dotted lines connect the points of wavelength specific critical scattering angles (Y1, Y2) from which the material specific scattering length densities are calculated. The specular beam position (S) is clearly distinct from the Yoneda peak positions. The area shielded by the beam stop is ruled in dark gray.

S12. Calculation of porosity and backfilling efficiency

The following equation states the relation between the critical angle α_c, the neutron wavelength λ, and the material specific scattering length density (SLD) (Kaune *et al.*, 2010):

$$\frac{\alpha_c}{\lambda} = \left(\frac{SLD}{\pi}\right)^{\frac{1}{2}}.$$

Through plotting of α_c vs. λ and extraction of the slope via linear regressions, $\left(\frac{SLD}{\pi}\right)^{\frac{1}{2}}$ is obtained and accordingly the SLD of the measured sample.

The porosity of the mesoporous titania is expressed by the following equation(Rawolle *et al.*, 2013):

$$\Phi = 1 - \frac{SLD_{por}}{SLD_{sol}},$$

where SLD_{por} is the measured neutron SLD of the mesoporous titania film and SLD_{sol} is the theoretical neutron SLD of a solid titania film ($\rho = 3.78\ \mathrm{g/cm^3}$) with a value of $SLD_{sol} = 2.349 \times 10^{-6}\ \text{Å}^{-2}$(Fu *et al.*, 2018).

The backfilling efficiency of material infiltrated into the mesoporous titania film is expressed as follows:

$$\xi = \frac{[SLD_{comp} - (1-\Phi)SLD_{sol}]}{SLD_{infil}},$$

where SLD_{comp} is the measured neutron SLD of a composite film of mesoporous titania and infiltrated material and SLD_{infil} is the theoretical neutron SLD of the infiltrated polymer or small molecule (Rawolle *et al.*, 2013).

S13. Remarks to the determination of SLDs

Due to the respective two Yoneda peaks (as-prepared titania and Si substrate or backfilled titania and Si substrate) lying close together, they cannot be distinguished by eye and their peak position is therefore determined by fitting a double Gaussian function into the vertical line cuts. By fitting one Gaussian for the silicon Yoneda Peak and one Gaussian for the as-prepared/backfilled titania into the vertical cuts, the material specific Yoneda peaks are determined as the peak centers of the single Gauss curves plus the angle corresponding to one detector pixel. This way, the determination of a Yoneda peak below the critical angle due to the maximum resolution of one pixel is avoided. The peak positions were determined with an accuracy of one pixel, represented by the error bars in angle. The wavelength dependent critical angles of the Si substrate were calculated from the literature Si density (Greenwood & Earnshaw, 1988) of $\rho = 2.336\ \mathrm{g/cm^3}$, resulting in a neutron SLD value of

$SLD_{Si} = 2.079 \times 10^{-6}$ Å^{-2} and were fixed as one wavelength dependent peak position during fitting of the double Gaussian into the vertical cuts.

S14. Detail of horizontal line cut modelling for one wavelength

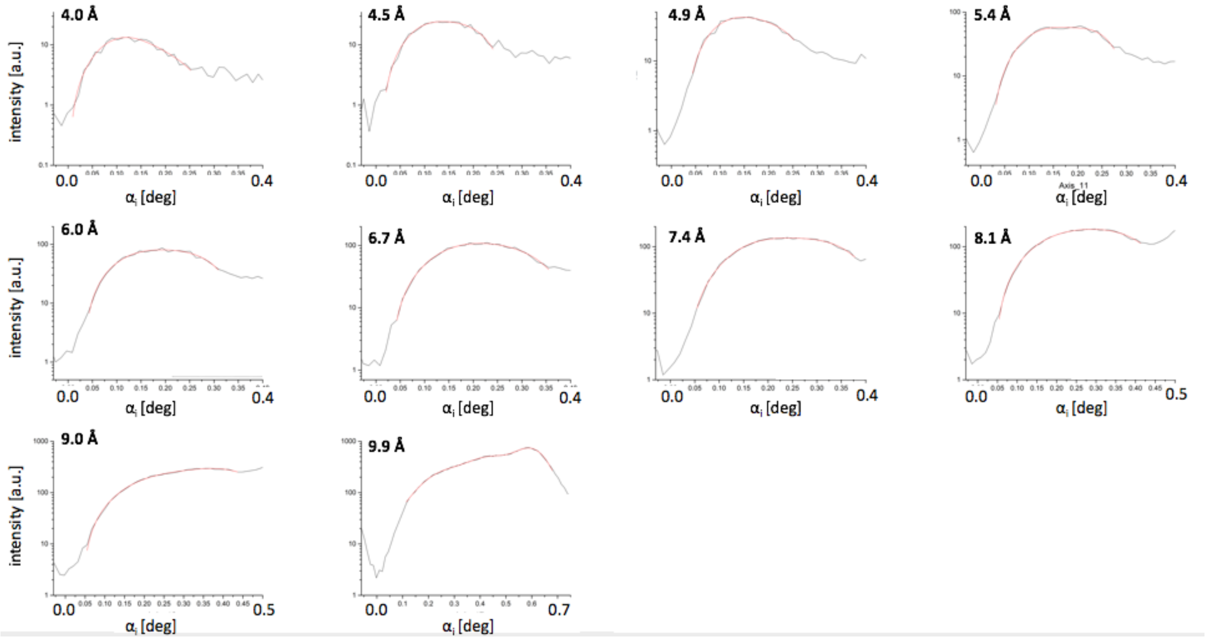

Figure S9 Vertical line cuts (black lines) of ToF-GISANS data and corresponding fits (red lines) to determine the Yoneda Peak positions of as-prepared titania films.

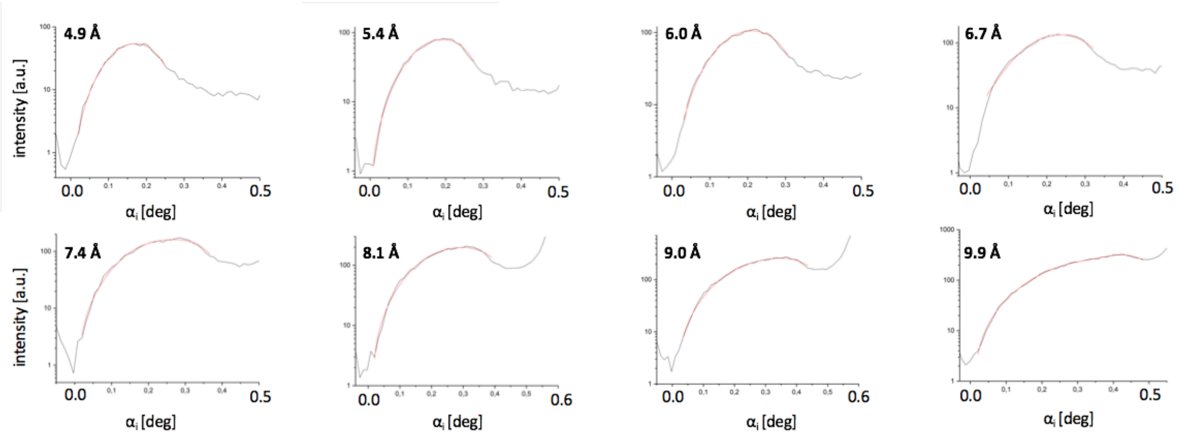

Figure S10 Vertical line cuts (black lines) of ToF-GISANS data and corresponding fits (red lines) to determine the Yoneda Peak positions of **PTB7-Th** backfilled titania films.

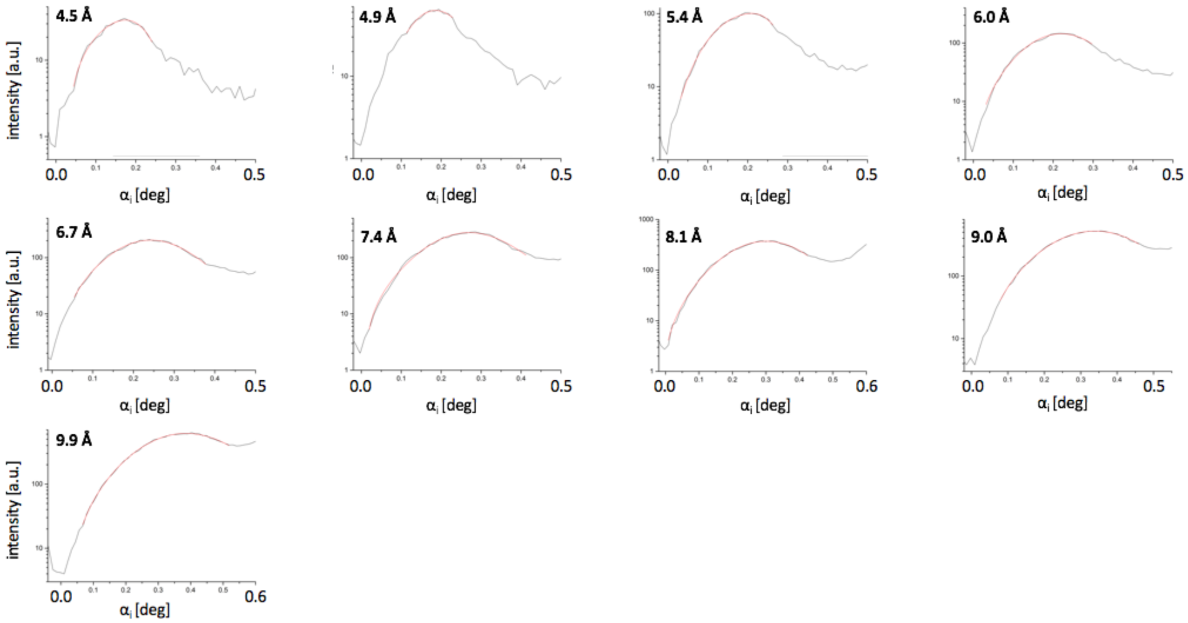

Figure S11 Vertical line cuts (black lines) of ToF-GISANS data and corresponding fits (red lines) to determine the Yoneda Peak positions of **Phen-Te-BPinPh** backfilled titania films.

S15. Horizontal line cut modelling detail

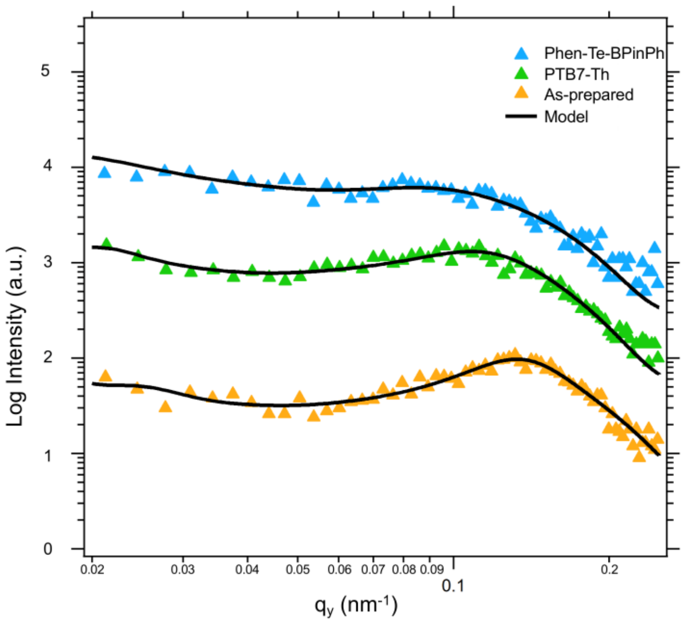

Figure S12 Detail of the main feature of the horizontal line cuts for the as-prepared mesoporous titania film (orange), **PTB7-Th** infiltrated titania (green), and **PhenTe-BPinPh** infiltrated titania (blue) for a neutron wavelength of 6.0 Å. Solid lines represent the best obtained model fit to the data and curves are shifted along the intensity axis for clarity.

Supporting References

Attwood, D. (1999). Soft X-Rays and Extreme Ultraviolet Radiation: Principles and Applications Cambridge: Cambridge University Press.

Chantler, C. T., Olsen, K., Dragoset, R. A., Chang, J., Kishore, A. R., Kotochigova, S. A. & Zucker, D. S. (2005). X-Ray Form Factor, Attenuation and Scattering Tables (version 2.1).

Fu, W., Li, G., Wang, Y., Zeng, S., Yan, Z., Wang, J., Xin, S., Zhang, L., Wu, S. & Zhang, Z. (2018). *Chem. Commun.* **54**, 58–61.

Greenwood, N. N. & Earnshaw, A. (1988). Chemie der Elemente Weinheim: VCH.

Hupf, E., Tsuchiya, Y., Moffat, W., Xu, L., Hirai, M., Zhou, Y., Ferguson, M. J., McDonald, R., Murai, T., He, G. & Rivard, E. (2019). *Inorg. Chem.* **58**, 13323–13336.

Kaune, G., Haese-Seiller, M., Kampmann, R., Moulin, J. F., Zhong, Q. & Müller-Buschbaum, P. (2010). *J. Polym. Sci. Part B Polym. Phys.* **48**, 1628–1635.

Song, L., Wang, W., Pröller, S., Moseguí González, D., Schlipf, J., Schaffer, C. J., Peters, K., Herzig, E. M., Bernstorff, S., Bein, T., Fattakhova-Rohlfing, D. & Müller-Buschbaum, P. (2017). *ACS Energy Lett.* **2**, 991–997.

Müller-Buschbaum, P. (2003). *Eur. Phys. J. E.* **12**, 443–448.

Müller-Buschbaum, P. (2013). *Polym. J.* **45**, 34–42.

Rawolle, M., Sarkar, K., Niedermeier, M. A., Schindler, M., Lellig, P., Gutmann, J. S., Moulin, J. F., Haese-Seiller, M., Wochnik, A. S., Scheu, C. & Müller-Buschbaum, P. (2013). *ACS Appl. Mater. Interfaces*. **5**, 719–729.

Roe, R.-J. (2000). Methods of X-ray and Neutron Scattering in Polymer Science Oxford University Press, Inc.

Rauch, H. & Waschkowski, W. (2003). *Neutron Data Booklet*, Vol. edited by A.-J. Dianoux & G. Lander, p. Grenoble: Institut Laue-Langevin.
